# Elevated ad libitum alcohol consumption following continuous theta burst stimulation to the left-dorsolateral prefrontal cortex is partially mediated by changes in craving

**DOI:** 10.3758/s13415-021-00940-7

**Published:** 2021-08-19

**Authors:** Adam M. McNeill, Rebecca L. Monk, Adam W. Qureshi, Stergios Makris, Valentina Cazzato, Derek Heim

**Affiliations:** 1 School of Social Sciences, Birmingham City University, 4 Cardigan Street, Birmingham, B4 7DB UK; 2 Department of Psychology, Edge Hill University, St Helens Road, Ormskirk, L39 4QP UK; 3 School of Psychology, Liverpool John Moores University, Byrom Street, Liverpool, L3 3AF UK; 4 Centre for Alcohol Research, Liverpool Health Partners, Liverpool, UK

**Keywords:** Inhibitory control, Attentional bias, Craving, TMS, AlcoholBinge drinking

## Abstract

Previous research indicates that following alcohol intoxication, activity in prefrontal cortices is reduced, linking to changes in associated cognitive processes, such as inhibitory control, attentional bias (AB), and craving. While these changes have been implicated in alcohol consumption behaviour, it has yet to be fully illuminated how these frontal regions and cognitive processes interact to govern alcohol consumption behaviour. The current preregistered study applied continuous theta burst transcranial magnetic stimulation (cTBS) to examine directly these relationships while removing the wider pharmacological effects of alcohol. A mixed design was implemented, with cTBS stimulation to right and left dorsolateral prefrontal cortex (DLPFC), the medial orbital frontal cortex (mOFC) and Vertex, with measures of inhibitory control, AB, and craving taken both pre- and post-stimulation. *Ad libitum* consumption was measured using a bogus taste task. Results suggest that rDLPFC stimulation impaired inhibitory control but did not significantly increase *ad libitum* consumption. However, lDLPFC stimulation heightened craving and increased consumption, with findings indicating that changes in craving partially mediated the relationship between cTBS stimulation of prefrontal regions and *ad libitum* consumption. Medial OFC stimulation and AB findings were inconclusive. Overall, results implicate the left DLPFC in the regulation of craving, which appears to be a prepotent cognitive mechanism by which alcohol consumption is driven and maintained.

## Introduction

Numerous humorous memes circulating on the internet poke fun at the notion of “just going for one drink” by documenting how planned moderate consumption of alcohol can, at times, escalate. Theoretically, alcohol-related cognitions, such as inhibitory control (Weafer & Fillmore, 2008), attentional bias (AB; see Field & Cox, 2008), and craving (Rose & Grunsell, 2008), have been identified as influences on people’s ability to curtail their alcohol consumption. These accounts have tended to place AB (Franken, 2003; Tiffany, 1990; Tiffany & Conklin, 2000) at the heart of explanations of addiction, and empirical work has examined how AB affects inhibitory control (Leung et al., 2017) and craving (Field et al., 2009). However, empirical research and theoretical contributions to date have painted a mixed picture as to how these cognitive mechanisms interact. On the one hand, research indicates that impairments of inhibitory control heighten AB. Conversely, studies also suggest that drug-related AB impairs inhibitory control (Leung et al., 2017). These accounts further point to a link between AB and craving, with elevated craving driving increases in AB and vice versa (Franken, 2003; Tiffany, 1990). Research, however, has tended to rely on alcohol administration techniques, which make it difficult to unpick the relative contributions of each of these processes. This also limits our ability to understand how their potential interactions drive consumption. Specifically, alcohol has been shown to exert widespread neuropsychopharmacological effects, such that whilst low doses activate the dopaminergic “reward” system in the dorsal striatum, higher doses appear to inhibit activity in prefrontal brain regions associated with executive functioning (Volkow et al., 2016). Research therefore is required to isolate cognitive changes from those wider effects of alcohol in order to ascertain their respective contributions to consumptive behaviours.

The literature documents a close relationship between inhibitory control and AB with a recent meta-analysis finding a small but significant positive relationship between inhibitory control and attentional processes (Leung et al., 2017). However, the direction of causation is in need of further elucidation. Previous findings suggest that alcohol impairs the ability to exert control over responses to alcohol-related stimuli (Adams et al., 2013), whereas others find that the presence of alcohol-related stimuli can be associated with in higher levels of inhibitory control impairments (Monk et al., 2017) and that elevated levels of AB and inhibitory control impairments predict consumption (Roberts et al., 2014). In short, it appears that attentional and inhibitory processes are interwoven; however, the relationship appears to be complex and multifaceted. It also is possible that the association between inhibitory control and AB may hinge on salience, whereby relevant cues may “grab” attention and, in turn, result in increased inhibitory control impairments, which can result in a diminished ability to exert control over responses to salient stimuli (Wilcockson & Pothos, 2015). It seems plausible that there may be a cyclical relationship between inhibitory control and AB in how these processes govern appetitive behaviours.

Research also has examined the extent to which fluctuations in inhibitory control may mediate the association between the initial exposure to alcohol and successive alcohol consumption (Field et al., 2010; Jones et al., 2013). In partial support of this hypothesis, Weafer and Fillmore (2008) found a correlation between inhibitory control impairments and drinking in a subsequent session, although others have found no direct association between transient changes in inhibitory control and successive consumption in the laboratory (Christiansen et al., 2013). Moreover, in a recent Ecological Momentary Assessment study, daily fluctuations in inhibitory control were associated of daily consumption (more so than prior planned consumption), while daily craving and implementation intentions appeared to be better predictors of drinking patterns throughout the period of study (Jones et al., 2018). Taken together, this body of work suggests that fluctuations in inhibitory control may not be as central in the maintenance of drinking behaviour as previously suggested. Instead, the effect of inhibitory control may be exerted through its interaction with AB and/or craving. In order to assess this assertion, research aided by methodological approaches that can isolate respective cognitive processes from alcohol’s wider pharmacological effects is required.

The complex relationship between inhibitory control, AB, and craving may be explained at the neural level, with research implicating adjacent prefrontal brain regions in both impulse control and salience attribution (Volkow et al., 2016). The Orbital Frontal Cortex (OFC), including the medial OFC (mOFC), has been shown to be related to salience attribution of potentially rewarding stimuli, including drugs and food (Volkow et al., 2013). Furthermore, AB for alcohol-related stimuli is associated with increased motivations to drink (Fadardi & Cox, 2008), heightened craving for food (Wang et al., 2004), and other drugs (Blum et al., 2012; Volkow et al., 2013) and also has been linked to increases in OFC activation (Volkow et al., 2016). The DLPFC also have been widely implicated in the maintenance and regulation of drug-seeking behaviour and particularly in wider substance-related executive functioning (Zilverstand, Huang, Alia-Klein, & Goldstein, 2018). Specifically, DLPFC have been associated with various components of inhibitory control (Robbins, Gillan, Smith, de Wit, & Ersche, 2012), and a moderating role has been suggested with regards to the DLPFC in substance-related craving (George & Koob, 2013). Nevertheless, as research has increasingly documented the neurological underpinnings of these processes, traditional imaging techniques have been hampered by their ability to elucidate causal links. Deploying neuromodulation techniques is required to examine the role of the DLPFC in the cognitive mechanisms implicated in initiating and sustaining substance-use behaviours.

Repetitive transcranial magnetic stimulation (rTMS) has increasingly been utilised as a tool to examine associated links between focal brain regions and specific cognitive processes and behaviours. For instance, research has investigated the role of prefrontal cortices in inhibitory control processes (Lowe et al., 2018). rTMS to the right dorsolateral prefrontal cortex (rDLPFC), for example, has been found to impair inhibitory control and to increase *ad libitum* alcohol consumption, although transient changes in inhibitory control do not appear to be directly associated with consumption (McNeill et al., 2018). Similarly, rTMS of the left-DLPFC was shown to induce inhibitory control impairment (as measured by the Stroop task), as well as increase food-related craving and consumption (Lowe et al., 2014), suggesting that lDLPFC is potentially important in appetitive regulation. More recently, research using rTMS indicates that lDLPFC may play a moderating role in craving, by reducing activation in the nucleus accumbens and mOFC (Li et al., 2017). While not examined in alcohol behaviours to date, Li and colleagues found that activation stimulation (relative to sham) in smokers resulted in lower levels of cue-induced craving, supplying evidence of a complex interplay between prefrontal regions in the regulation of consumption behaviours.

This preregistered study (osf.io/hjy4n) applied a randomised mixed design to transiently inhibit the neural structures associated with AB, inhibitory control, and craving (DLPFC, mOFC) to illuminate how these processes interact and drive consumption. In accordance with previous findings (McNeill et al., 2018), it was hypothesised that stimulation to the DLPFC will impair inhibitory control, while stimulation of the mOFC would significantly reduce AB for alcohol-related cues in a manner akin to observations in smokers (Li et al., 2017). As previously indicated (Adams et al., 2013; Monk et al., 2017), it was expected that inhibitory control impairments will, in turn, increase alcohol-related AB. Furthermore, stimulation to the lDLPFC was expected to result in increased alcohol-related craving and to increase AB, in a manner akin to observations of wider appetitive behaviours (Lowe et al., 2018). Finally, heightened *ad libitum* alcohol consumption was hypothesised to be observed post right- and left-DLPFC stimulation, but not following mOFC, in accordance with appetite research (Lowe et al., 2018).

## Methods

### Participants

Eighty participants aged 18 to 23 years (*M*_*age*_ = 20.38, *SD* = 2.79, 44 males) were recruited via digital advertising within a University in the United Kingdom. To be eligible, participants were required to be aged between 18 and 49 years due stimulation protocol guidelines, regularly exceed the 14 UK units weekly recommendation and speak fluent English. Before taking part participants underwent medical screening due to the risks associated with TMS, although these risks are considered to be very minimal if screened correctly (Rossi et al., 2009). Participants were prohibited from taking part in instances where medical screening indicated any neurological risk factors, syncopy, drugs active in the central nervous system (e.g., antipsychotics, antidepressants, or recreational stimulants) and low levels of sleep of the previous night (Rossi et al., 2009; Wassermann, 1998). Furthermore, participants who specified a personal or family history of problematic alcohol use also were excluded. Participants either received course credit or £10 as a means of reimbursing them for their time. The study received ethical review and clearance from the University’s Department of Psychology Research Ethics Committee

### Design

A mixed design was employed; the between-participants independent variable was the brain region stimulated. Participants were randomly allocated to one of four stimulation region conditions; rDLPFC (*n* = 20), lDLPFC (*n* = 20), mOFC (*n* = 20), or Vertex (*n* = 20). Measures of subjective craving, inhibitory control, and attentional bias were taken both pre- and post-stimulation, followed by an *ad libitum* consumption task.

### Materials

#### *Time Line Follow Back* (TLFB: Sobell & Sobell, 1990)

Participants are required to retrospectively report their daily alcohol consumption (in units) for the previous 14 days.

#### Alcohol Use Disorder Identification Test (AUDIT: Saunders, Aasland, Babor, & la Fuente de, 1993)

The AUDIT is a 10-item questionnaire concerning levels of alcohol consumption and its consequences. Scores range from 0-40, with scores ≥8 representative of alcohol consumption of a hazardous level. Reliability analysis demonstrated high internal consistency in the current sample (*α* = 0.82).

#### Barrett Impulsivity Scale (BIS-11: Patton et al., 1995

The BIS is a multidimensional scale, consisting of three subscales; attentional, motor, and nonplanning impulsiveness. BIS-11 includes 30 fixed response items (e.g., I plan tasks carefully), which are assessed on a 4-point scale (rarely/never – almost always/always). Higher scores are indicative of increased impulsivity. The attentional (*α* = 0.66) and motor (*α* = 0.64) subscales showed acceptable reliability, nonplanning (*α* = 0.75), demonstrating good reliability and overall BIS-11 (*α* = 0.82), displaying high reliability.

#### Desire for Alcohol Questionnaire – brief form (DAQ; Love et al., 1998)

The DAQ is a 14-item, four-dimensional alcohol craving the factors include positive and negative reinforcement, strong desires and intentions, and mild desires and intentions. The scale is scored on 1-7 Likert scale with higher scores indicative of higher craving. Reliability analysis revealed the DAQ to reliable both pre- (*α* = 0.81) and post- (*α* = 0.79) stimulation.

#### Mood Scale

The scale consisted of 6 statements (e.g., I feel happy, I feel sad) to which participants responded on a 100-mm Visual Analogue Scale ranging from “Not at all” to “Extremely.”

#### Behavioural tasks

##### Stop-signal task (SST: Verbruggen et al., 2008)

The Stop-Signal task consists of two concurrent tasks: a go task (75% of trials), which is a choice reaction task where participants categorise arrows on the screen based on their orientation (left or right), and a stop task (25% of trials) where an auditory tone (the stop signal) indicates that participants should inhibit their response to the go signal. Participants are required to respond as quickly and accurately as possible to the stimuli with a predetermined corresponding key. Upon hearing the auditory tone (the stop signal), participants are required to inhibit their response. After 2,000 ms, the trial will time out.

On the stop trials, tones are delivered at fixed delays (known as stop-signal delays [SSD]) of between 50 ms and 500 ms following the presentation of the go stimulus. The stop-signal task uses these SSDs dynamically, based on participant performance. The *one-up one-down tracking procedure* (Logan et al., 1997) was implemented, which adjusts the SSDs after each trial. After successful inhibition trials, the SSD increases by 50 ms, handicapping the stop signal process on the next stop signal trial. Unsuccessful inhibition trials result in the SSD decreasing by 50 ms. In accordance with the “horse race” model, the degree of difficulty in inhibiting responding increases as the delay between the go stimulus and the stop signal increases (Logan et al., 1984). Providing an outcome variable of stop-signal reaction time (SSRT), calculated using the integration method (Verbruggen & Logan, 2009). This comprises of subtracting the mean SSD value from the n^th^ reaction time. This is calculated by ranking the reaction times from the fastest to slowest, then multiplying the number of GoRTs (144 in this instance) by the proportion of inhibition errors. For example, if a participant made 50% inhibition errors, the 72^nd^ fastest RT would be n^th^ values (144 x 0.50 = 72). Greater SSRT values are indicative of poorer inhibitory control. Reliability analysis indicated that the SST was reliable both pre- (*α* = 0.80) and post-beverage (*α* = 0.78). The SST was delivered using Millisecond Inquisit Lab version 4. Participants received 3 experimental blocks of 64 trials, allowing for a short break between each block, taking approximately 6 minutes to complete.

##### Visual Probe task (VPT; Schoenmakers et al., 2008)

The visual probe task was programmed in Experiment Builder and deployed in concurrence with the Eye-link 1,000 eye-tracker (SR Research, Mississauga, ON, Canada) to assess attentional-bias. The task begins with the presentation of a fixation cross, signalling the beginning of each trial. Following this manual submission of any key triggers the exhibition of images, presented side-by-side 60-mm apart in alcohol/neutral pairs. Each trail had a duration of 2,000 ms, and the task consisted of 40 trials in total. The reliability of the Visual Probe task was shown to be poor both pre- (*α* = 0.53) and post-stimulation (*α* = 0.36); however, this is consistent with previous findings (Field & Christiansen, 2021).

##### Gaze Contingency Task (Wilcockson & Pothos, 2015)

The gaze contingency task was programmed using Experimenter Builder software and delivered on an EyeLink Desktop 1,000 eye-tracker to measure inhibitory control for AB. Here, each trial presented a fixation target on the screen. Participants are instructed to focus their attention on the fixation target. Once participants have attended to the fixation target for a fixed interval of 1 second, a distractor stimulus will appear (only 1 per trial), either an alcohol-related or neutral image. If the participant looks at the distractor stimulus (i.e., if the participant's gaze was to leave the fixation target boundary), then the distractor stimulus will disappear instantly. Therefore, participants are unable to fixate upon the distractor stimuli. The distractor stimuli will only reappear once participants fixate on the fixation target again for 10 ms (i.e., less than 1 frame on a 60 Hz monitor). The fixation target will be displayed for 5 s in total, so the maximum duration for which a distractor stimulus will be displayed on the screen is 4 s. “Break frequency”—the number of times that participants attended peripherally presented stimuli—will be measured, producing a DV that is a direct measure of the level of distraction created by peripheral stimuli of different types.

### Theta Burst stimulation procedure

Continuous theta burst stimulation (cTBS) was performed using a 70-mm figure-of-eight stimulation coil (Magstim D70^2^ Coil), connected to a Magstim SuperRapid 2 Stimulator (The Magstim Company, Carmarthenshire, Wales). This produces a magnetic field of up to 0.8 T at the coil surface. To appropriately select the TMS stimulation intensity for each participant, the resting motor threshold (rMT) for the first dorsal interosseous muscle (FDI) of the participant’s dominant hand was visually determined (Pridmore et al., 1998). Here, the coil was positioned over the left or right motor cortex (for right or left-hand dominance respectively) in correspondence with the optimal scalp position (OSP). It was detected by moving the intersection of the coil in 1-cm steps around the motor hand area of the left motor cortex, while delivering TMS pulses at constant intensity. The rMT was defined as the lowest stimulus intensity able to evoke a visible finger twitch on at least five of ten trials.

cTBS was delivered over the rDLPFC, lDLPFC, and mOFC. The vertex was chosen as a control site to account for nonspecific effects of TMS. The approximate locations of the stimulating areas were identified on each participant's scalp by means of the international 10-20 EEG System Positioning (F4 – rDLPFC, F3 – lDLPFC, Fpz – mOFC, Cz – Vertex). In keeping with past research, for rDLPFC stimulation, the coil was positioned on the F4 location. Three-pulse bursts at 50 Hz repeated every 200 ms for 40 s were delivered at 80% of the subject’s rMT (equivalent to “continuous theta burst stimulation” cTBS; *M* = 48.68, *SD* = 7.96), resulting in 600 pulses in total (Huang et al., 2005). The coil was positioned tangentially to the scalp, at 90° from the midsagittal line, to modulate contralateral M1 excitability and interfere with cognitive functions. The coil was held by hand throughout stimulation and the exact coil position was marked by ink to ensure an accurate and consistent positioning of the coil throughout the experiment. TBS mimics the theta rhythm (4-8 Hz) to induce long-term potentiation of the NMDA receptors, reducing cortical excitability lasting up to 50 minutes (Cho et al., 2010; Huang et al., 2005). It is for this reason the cTBS protocol was adopted for the current study to provide a reliable effect and duration to complete experimental tasks.

### *Ad libitum* alcohol consumption

*Ad libitum* alcohol consumption was measured by means of the Bogus Taste test. Participants were presented with three different beers (330 ml each) and asked to rate them on several dimensions of taste (e.g., bitterness and sweetness). They were informed that they could consume as much or little as they liked to successfully complete the task. *Ad libitum* consumption is measured by subtracting the remaining volume from the initial volume.

### Procedure

As per ethical and risk assessment guidelines, participants interested in partaking in the study had to complete medical screening a minimum of 24 hour before any arranged session. This gave them opportunity to consult friends, family, or a health professional, or ask any questions of the researcher. Experimental sessions took place in University laboratories between 12 and 6 pm. Before the study session commenced, participants were required to provide a breathalyser reading of 0.00 mg/l (Lion Alcolmeter 400, Lion Laboratories, Vale of Glamorgan, United Kingdom), confirm that they had not consumed excessive caffeine, and had adequate sleep the night previous. A battery of questionnaires was then completed (TLFB, AUDIT, BIS-11, DAQ, mood scale), followed by baseline SST and VPT. Participants were then randomly allocated to a stimulation condition and received cTBS to associated brain region according to the protocol. Once the cTBS was completed participants repeated the DAQ, mood scale, SST, and VPT in a counterbalanced order, taking approximately 15 minutes. Finally, participants completed the bogus taste task and were fully debriefed on completion.

### Results

#### Demographics and baseline measures

A MANOVA was performed to assess if any differences in baseline measures (TLFB, AUDIT, BIS, and rMT) between conditions were present. Findings indicated that no significant differences between conditions *Wilks’ Lambda* = 0.72, *F*(12, 199.16) = 1.63, *p* = 0.07, *η*$\frac{2}{p}$ = 0.10, as such none of these measures were taken forward into the main analysis as covariates. See Table 1 for means and standard deviations.

**Table 1:** Means and standard deviations for demographics and baseline measures

	Mean	SD
Age*	20.38	2.79
AUDIT	9.51	4.44
TLFB	29.41	28.90
BIS	58.19	11.48
rMT (%)	60.85	10.85

AUDIT = Alcohol Use Disorder Identification Test, TLFB = Timeline Follow Back, BIS = Barratt Impulsivity Scale, rMT = Resting Motor Threshold. *Ages ranged from 18 to 23 years.

#### Subjective mood ratings

The influence of stimulation on mood ratings was assessed using two (one for positive and one for negative mood ratings, 2 (time; pre- and post-stimulation) x 4 (condition; rDLPFC, lDLPFC, mOFC, and Vertex) mixed ANOVAs. No effect of time *F*(1, 76) = 0.50, *p* = 0.48, *η*$\frac{2}{p}$ = 0.007 or time x condition interaction *F*(3, 76) = 1.92, *p* = 0.13, *η*$\frac{2}{p}$ = 0.07 was observed for positive mood ratings. Neither was there an effect of time *F*(1, 76) = 0.13, *p* = 0.72, *η*$\frac{2}{p}$ = 0.002, or time x condition interaction *F*(3, 76) = 1.05, *p* = 0.38, *η*$\frac{2}{p}$ = 0.04 for negative mood state ratings. This indicates that stimulation does not appear to alter the mood of participants, eliminating mood as potential explanation for changes in cognitive performance and *ad libitum* consumption.

#### Inhibitory Control

**Fig. 1:**
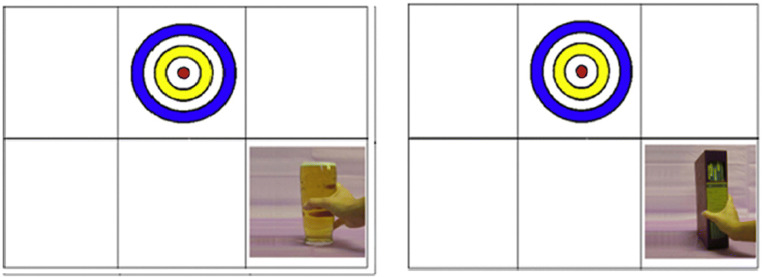
Taken from Wilcockson and Pothos (2015). Example of the presentation of alcohol-related (left) and neutral stimuli (right)

**Fig. 2:**
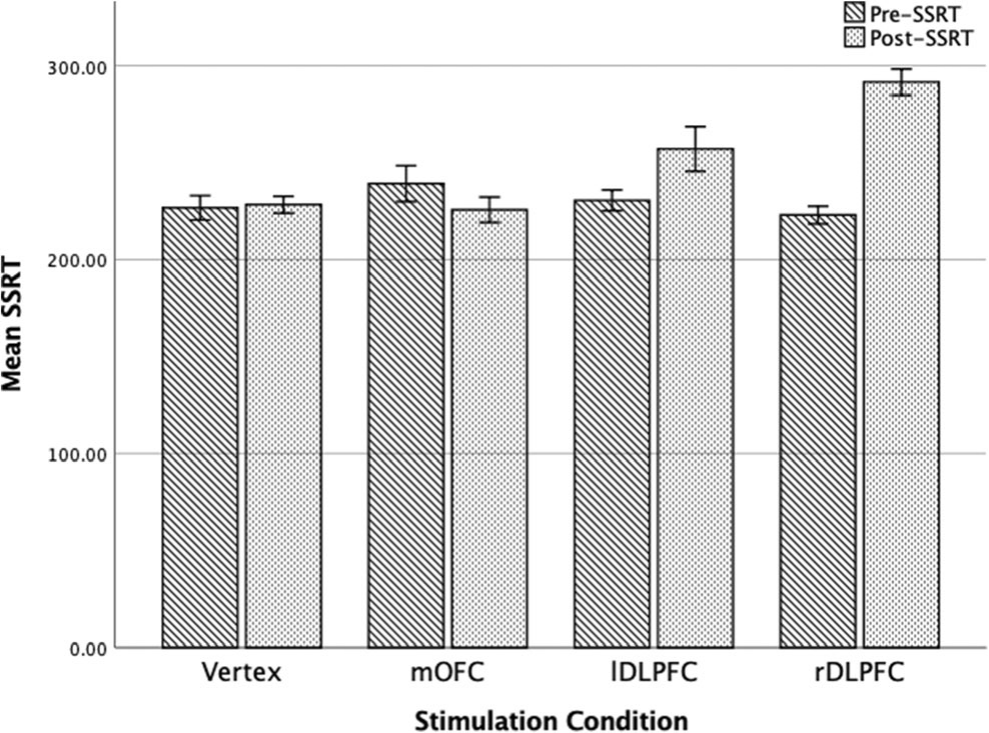
Pre- and post- stimulation mean and standard error inhibitory control (SSRT) scores for each stimulation condition.

A 2 x 4 mixed ANOVA was undertaken to assess the effects of stimulation on SSRT, with time as the with participants variable (pre- and post-SSRT) and stimulation condition as the between variable (rDLPFC, lDLPFC, mOFC, and Vertex). There was a significant difference beween pre- and post-SSRT score *F*(1, 76) = 24.36, *p* < 0.001, *η*$\frac{2}{p}$ = 0.24. The ANOVA also revealed a significant time x condition interaction *F*(3, 76) = 18.11, *p* < 0.001, *η*$\frac{2}{p}$ = 0.42. Bonferroni corrected pairwise comparisons indicated that SSRT scores significantly increased following rDLPFC (*p* < 0.001) and lDLPFC (*p* < 0.01), demonstrating inhibitory control impairments. No significant differences were revealed between pre- and post-SSRT scores for mOFC (*p* = 0.11) and Vertex (*p* = 0.85). For means and standard error see Figs. 1 and 2.

#### Craving

**Fig. 3:**
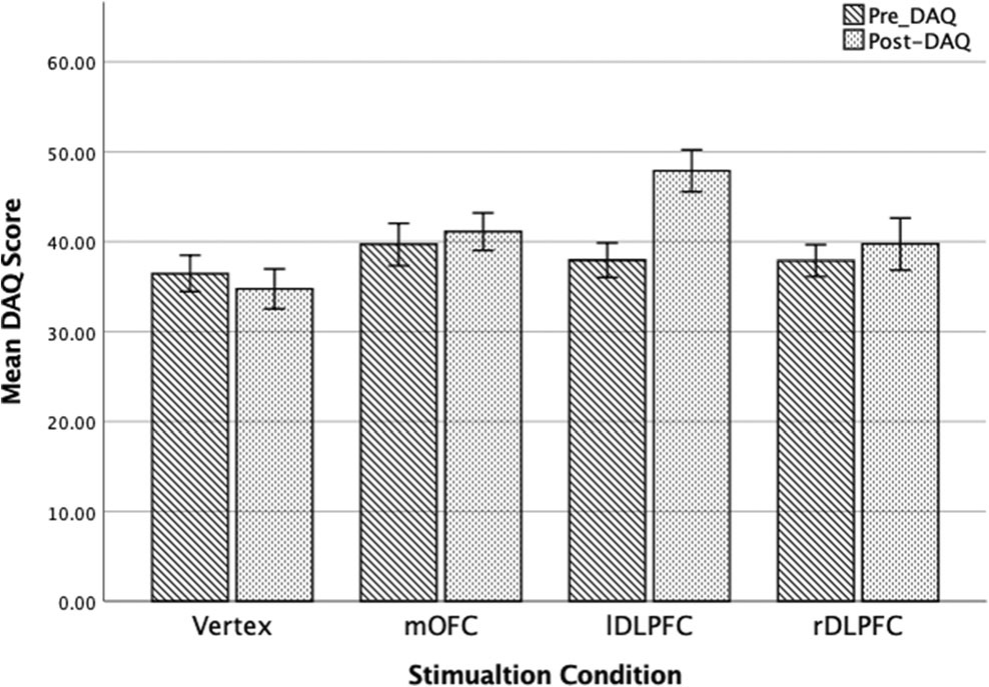
Pre- and post- stimulation mean and standard error craving (DAQ) scores for each stimulation condition

A 2 (time; pre- vs. post-stimulation) x 4 (condition; rDLPFC, lDLPFC, mOFC, and Vertex) mixed ANOVA was used to examine the relationship be modulation of prefrontal regions and alcohol-related craving. There was a significant effect of time *F*(1, 76) = 12.83, *p* < 0.01, η$\frac{2}{p}$ = 0.14, indicating an overall increase in craving following stimulation. More pertinently, a significant time x stimulation condition was detected *F*(3, 76) = 9.57, *p* < 0.001, *η*$\frac{2}{p}$ = 0.27, with Bonferroni corrected pairwise comparisons indicating that craving significantly increased from baseline following stimulation to the lDLPFC (*p* < 0.001). Craving did not increase following stimulation to any other brain region (rDLPFC *p* = 0.25, mOFC *p* = 0.38, Vertex *p* = 0.29). For means and standard errors see Fig. 3.

#### Attentional Bias

**Fig. 4:**
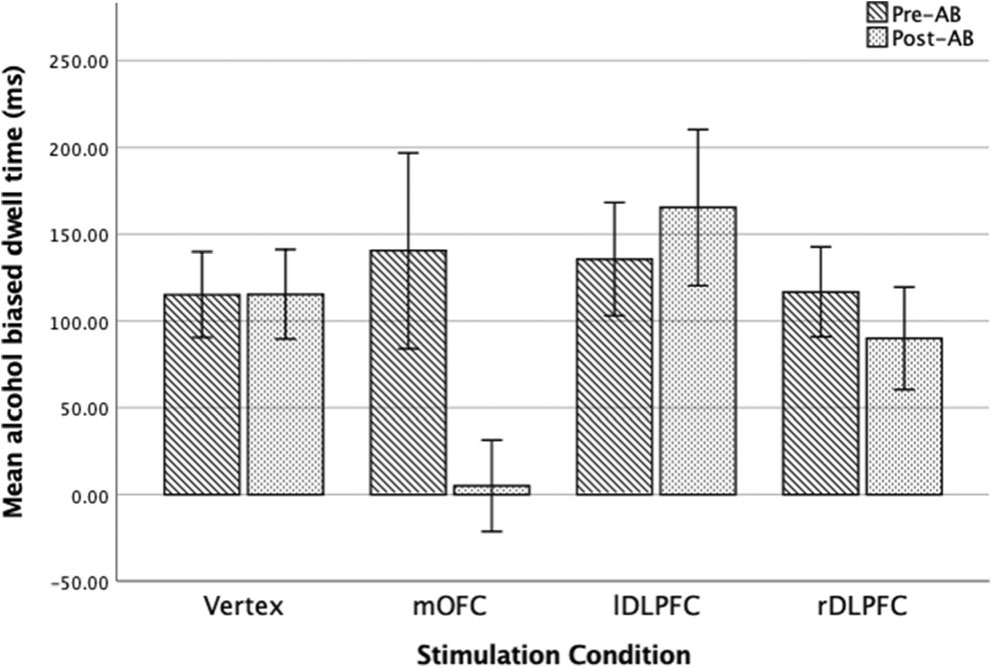
Means and standard errors for attentional bias dwell time, pre- and post-stimulation, following each stimulation condition.

**Fig. 5:**
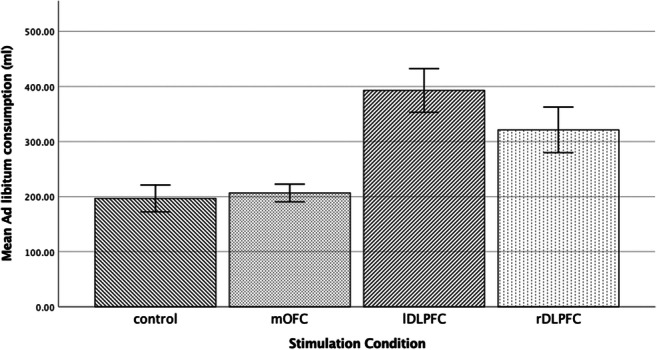
Means and standard errors for ad libitum alcohol consumption following each stimulation condition

For greater clarity and ease of interpretation a single value was calculated for pre- and post-AB, subtracting the values for neutral dwell time from alcohol cue dwell time (Weafer & Fillmore, 2013). A 2 (time; pre- vs. post-stimulation) x 4 (condition; rDLPFC, lDLPFC, mOFC, and Vertex) mixed ANOVA was used to examine the relationship between modualation of prefrontal regions and AB. There was a significant time x condition interaction, *F*(3, 76) = 3.98, *p* < 0.025, η$\frac{2}{p}$ = 0.14. While Bonferroni corrected pairwise comparisons revealed that there was a significant decrease in AB following stimulation to the mOFC (*p* < 0.001), there was no other significant changes in AB for other stimulation conditions (rDLPFC *p* = 0.46, lDLPFC *p* = 0.41, Vertex *p* < 0.999). This suggests that stimulation to the mOFC impairs the saliency processing of alcohol-related cues, resulting in the diminishment of AB. See Figs. 4 and 5 for means and standard errors.

#### Gaze Contingency Task

A series of 2 (cue; alcohol vs. neutral) x 2 (time; pre- vs. post- stimulation) x 4 (condition; rDLPFC, lDLPFC, mOFC, and Vertex) mixed ANOVAs were used to assess the effects of stimulation on inhibitory control for AB. Overall “break frequency” for each cue type indicated no effect of cue type, time, or condition interactions (all *p’s* > 0.07). Previous research has found that distractor stimuli further away from the fixation target significantly increases “break frequency” rate (Qureshi, Monk, Pennington, Wilcockson & Heim, 2019). Hence, two more ANOVAs were used to assess “near” and “far” stimuli. Findings for near were the same as overall, indicating no significant effects (all *p’s* > 0.05). However, for far, there was significant effect of cue x time interaction *F*(1, 76) = 6.13, *p* < 0.025, *η*$\frac{2}{p}$ = 0.08; however, this was significantly greater for neutral compared to alcohol-related stimuli. No other significant effects or interactions were observed (all *p* > 0.21).

#### Ad libitum consumption

A univariate ANOVA was used to evaluate the influence of stimulation condition on *ad libitum* consumption, demonstrating a significant effect *F*(3, 76) = 9.35, *p* < 0.001, *η*$\frac{2}{p}$ = 0.27. Bonferroni corrected pairwise comparisons revealed that *ad libitum* consumption was considerably greater following stimulation to the lDLPFC compared with mOFC (*p* < 0.001) and vertex (*p* < 0.001), and consumption post rDLPFC compared with vertex was significantly higher (*p* < 0.05). There was significant differences between stimulation of the right and left DLPFC (*p* = 0.72), rDLPFC and mOFC (*p* = .08), plus mOFC and Vertex (*p* < 0.999). See Figures 4 and 5 for means and standard errors.

#### Mediation Analyses

**Fig. 6:**
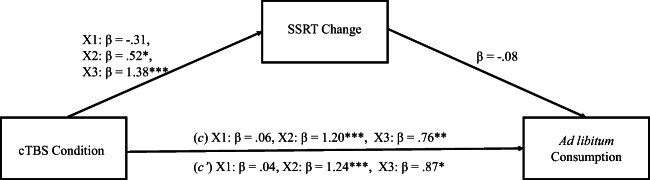
Mediation model assessing impairments in inhibitory control as a mediator between stimulation condition and ad libitum consumption. *p < 0.05; **p < 0.01; ***p < 0.001

Mediation analysis was undertaken using the PROCESS 3.4 macro for SPSS to assess whether impairments in inhibitory control mediate the relationship between cTBS condition and *ad libitum* consumption. First, a variable representing impairments of inhibitory control was computed by subtracting the prestimulation SSRT value from the poststimulation SSRT values. Greater SSRT change values indicated greater impairments of inhibitory control. With use of the multicategorical function in PROCESS 3.4, dummy variables were formed, comparing each condition to control (Vertex; X1 = mOFC vs. Vertex, X2 = lDLPFC vs. Vertex, X3 = rDLPFC vs. Vertex). First, there was a significant direct effect of stimulation condition on *ad libitum* consumption (*c*_*1*_ pathway) *F*(3, 76) = 8.63, *p* < 0.001, *R*^*2*^ = 0.25, X2 *t*(76) = 4.31, *p* < 0.001, 95% confidence interval (CI) [105.60, 286.70], X3 *t*(76) = 2.74, *p* < 0.01, 95% CI [34.10, 215.20]; however, the mOFC stimulation did not show elevated consumption X1 *t*(76) = 0.22, *p* = 0.83, 95% CI [−80.60, 100.60]. Overall, path *a* demonstrated a significant effect of stimulation on SSRT *F*(3, 76) = 18.11, *p* < 0.001, *R*^*2*^ = 0.42, with both left and right DLPFC stimulation conditions predicting increases in SSRT, X2 *t*(76) = 2.10, *p* < 0.05, 95% CI [1.24, 48.70], X3 *t*(76) = 5.62, *p* < 0.001, 95% CI [43.18, 90.64]; however, mOFC stimulation did not X1 *t*(76) = 1.27, *p* = 0.21, 95% CI [−38.85, 8.61]. The overall mediation model was significant *F*(4, 75) = 6.51, *p* < 0.001, *R*^*2*^ = 0.26, SSRT change but did not predict *ad libitum* consumption (*b* path) *t*(75) = 0.60, *p* = 0.55, 95% CI [−1.14, 0.61]. The *c* pathway, however, remained significant for lDLPFC stimulation X2 *t*(75) = 4.32, *p* < 0.001, 95% CI [109.16, 296.25] and rDLPFC X3 *t*(75) = 2.62, *p* < 0.025, 95% CI [34.01, 250.41], and mOFC remained nonsignificant X1 t(75) = .13, *p* = 0.90, 95% CI [−85.94, 97.90], indicating that SSRT change did not act as a mediator. See Fig. 6 for mediation model.

**Fig. 7:**
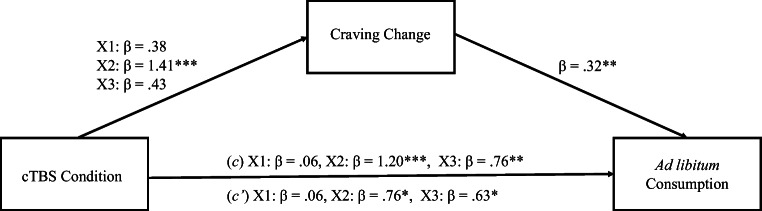
Mediation model examining changes in craving as a mediator between stimulation condition and ad libitum consumption. *p < 0.05; **p < 0.01; ***p < 0.001

A second Mediation analysis was undertaken using the PROCESS 3.4 macro for SPSS to investigate craving as a mediator between stimulation and *ad libitum* consumption. As above, a variable representing changes in craving associated with stimulation was computed by subtracting the prestimulation DAQ value from the poststimulation DAQ values, with higher change values indicative of heighten craving. As previous, the multicategorical function in PROCESS 3.4 was used to compute dummy variables, comparing each condition to control (Vertex; X1 = mOFC vs. Vertex, X2 = lDLPFC vs. Vertex, X3 = rDLPFC vs. Vertex). The *c* path remained consistent with the previous mediation model. Overall, path *a* demonstrated a significant effect of stimulation on craving *F*(3, 76) = 9.57, *p* < 0.001, *R*^*2*^ = 0.27, with stimulation of the lDLPFC associated with significant elevations in craving, X2 *t*(76) = 5.13, *p* < 0.001, 95% CI [7.12, 16.17], however, mOFC stimulation X1 *t*(76) = 1.37, *p* = 0.18, 95% CI [−1.42, 7.63] and rDLPFC X3 *t*(76) = 1.56, *p* = 0.12, 95% CI [−0.98, 8.07]. The overall mediation model was significant *F*(4, 75) = 9.09, *p* < 0.001, *R*^*2*^ = 0.33, with changes in craving significantly predicting *ad libitum* consumption (*b* path) *t*(75) = 2.83, *p* < 0.01, 95% CI [1.86, 10.60]. The *c* pathway, however, remained significant for lDLPFC stimulation X2 *t*(75) = 2.45, *p* < 0.05, 95% CI [23.09, 224.10] and rDLPFC X3 *t*(75) = 2.32, *p* < 0.05, 95% CI [14.53, 190.57], whereas mOFC X1 t(75) = 0.21, *p* = 0.83, 95% CI [−97.11, 78.29] remained nonsignificant. These findings imply that craving only partially mediates the relationship between stimulation and continued *ad libitum* consumption. See Fig.7 for mediation model.

## Discussion

The current study applied cTBS to inhibit the neural structures associated with attentional bias, inhibitory control, and craving (DLPFC, mOFC), in order to illuminate how these processes interact and drive consumption. Findings can be summarised as follows: First, in accordance with our hypothesis, stimulation to the DLPFC resulted in impaired inhibitory control, while mOFC stimulation decreased alcohol-related AB. Second, impairments in inhibitory control resulting from DLPFC stimulation did not appear to be related to increases of alcohol-related AB. Although craving following lDLPFC was heightened, as anticipated, the predicted associated changes in the AB were not evident. However, while stimulation to the rDLPFC did not result in increases *ad libitum* consumption, as expected, drinking was heightened following lDLPFC stimulation. This increase appeared to be partially mediated by changes in self-reported craving.

Beginning with a discussion of null findings, we failed to identify a relationship between transient inhibitory control impairments and drinking maintenance or increased AB. The current findings therefore indicate that stimulation to the DLPFC impaired inhibitory control, consistent with previous stimulation research (Brevet-Aeby et al., 2016); however, these impairments were not found to mediate *ad libitum* consumption. This may suggest that while the DLPFC appears to modulate the extent to which individuals can exert control over prepotent responses, this capacity does not appear to be directly related to drinking behaviour. This contrasts with early suggestions by Field et al. (2010) but appears to be consistent with a growing body of more recent contributions (Christiansen et al., 2013; Jones et al., 2018; Jones et al., 2020; McNeill et al., 2018). Present (null) findings therefore appear to undermine further the notion of a causal link between inhibition impairments and loss of volitional control over actual beverage alcohol consumption and therefore may be more consistent with theoretical models that view inhibitory control as a more multifaceted construct that is embedded within wider cognitive processing networks (Verbruggen, 2016). To this effect, findings from wider inhibitory control measures that engage aspects of attentional control (Eriksen Flanker, Stroop) may have presented different findings similar to those observed in other appetitive behaviours (Lowe et al., 2017, 2018). Future research should attempt to unpack inhibitory control in the context of wider executive functions.

In a similar vein, the current study also failed to find a relationship between impairments in inhibitory control and AB. These results contrast with the suggestions that impaired inhibitory control may adversely impact people’s ability to control attention to alcohol-related cues (Adams et al. 2013) and that alcohol-related stimuli impairs impulse control (Monk et al., 2017). The current study showed reduced AB after mOFC stimulation. This however did not translate into the predicted decreases in inhibition failures as measured by the Gaze Contingency Task. Furthermore, the current findings did not yield any support for the relationship between AB and craving as previously theorised (Franken, 2003; Tiffany, 1990; Tiffany & Conklin, 2000). While stimulation to the lDPFC elevated alcohol-related craving, this did not appear to translate into increases in AB (in contrast with Lowe et al., 2018). When considered alongside meta-analyses indicating significant yet weak links between impulsivity and AB (Luenge et al., 2017) and between craving and AB (Field et al., 2009), the current research casts doubt on the notion of a simple (causal) relationship between these processes. Rather, any relationships appear likely to be nuanced and likely to be underpinned by a wider complex neural network (Koob, 2014), which require further research scrutiny.

Further evidence of the complexity of these processes is evidenced when turning to the finding that alcohol consumption was elevated following lDLPFC stimulation, although this increase appeared to be partially mediated by changes in craving. To our knowledge, this is the first study to attempt to examine the role that lDPFC plays in exerting control of alcohol consumption, extending previous findings in relation to wider appetitive behaviours (Lowe et al., 2018). Moreover, by isolating craving from wider pharmacological changes associated with consumption, this research adds weight to the notion that craving may represent an important cognitive mechanism through which drinking episodes are maintained (Rose et al., 2010). Indeed, it may be suggested that craving, rather than inhibitory control (which did not appear to mediate consumption) is a more central cognitive process through which consumption is initiated and maintained. In this way, our work may provide an explanation for why efforts to train inhibitory control have not proved efficacious for reducing consumption (Jones et al., 2016). Targeting craving therefore may be a more fruitful avenue for future exploration and may better inform interventions which seek to reduce the number of drinking episodes and may help minimise people’s sense of losing control.

The current findings should be viewed with caution in light of a number of potential limitations. First, while the current sample size is similar to other TMS research in this area (Lowe et al., 2017; Lowe et al., 2018), further explorations of this kind are encouraged, particularly when seeking to unpick further the interactions between processes (inhibitory control, AB and craving) where effects may be small (for instance, the relationship between craving and AB in substance users; Field, Munafò, & Franken, 2009). It is, however, worth noting that post-hoc power analysis revealed acceptable observed power for the current sample (1-β = 0.88). Second, it should be noted that the current study began testing responses immediately post stimulation to allow for competition of measures during the suggested 45-minute duration of stimulation effect (Huang et al., 2005). This has the benefit of reducing demand on participants and limits the procedural signalling which may occur where multiple stimulation sessions are utilised (i.e., one stimulation session for cognitive and questionnaire measures and a second identical stimulation for behavioural measures). It has been observed, however, that cTBS does not reach peak efficacy until around 14-40 minutes poststimulation (ibid), and, as such, it should be noted that there may have been resultant variability in observed cognitive and behavioural changes. A note of caution should be added with regards stimulation of the mOFC. This may be considered uncomfortable and may explain null findings, such as no elevation of *ad libitum* consumption. Finally, caution is needed when seeking to generalise the current findings, taken from a young student sample, to the populations where developmental differences may be expected in terms of prefrontal structures and impulse control. Specifically, it has been suggested that prefrontal brain regions and, consequently, impulse control continue to develop to the age of 25 years (Spear, 2013). Future research should be expanded to older populations to examine whether the current findings apply.

## Conclusions

The current study represents an initial attempt to use TMS to isolate changes in cognitive processes (inhibitory control, attentional bias, and craving) from wider pharmacological effects of alcohol. In so doing, it examined how reputedly important cognitive processes associated with alcohol behaviours interact and relate to alcohol consumption. In general, findings suggest while DLPFC may be important in the control of prepotent responses, such changes do not manifest in increased consumption. Likewise, while the lDPFC appears to exert a degree of control over craving processes, current findings did not support the notion that heightened craving is associated with elevations in alcohol-related attentional bias. Rather, the current findings suggest that craving may be a more central (mediatory) mechanism than inhibitory control and attentional bias in the self-regulation of alcohol consumption. While we advocate for further research to unpick the complex interaction between cognitive processes and their underlying neural substrates, we tentatively suggest that future interventions may benefit from increased consideration of craving as a significant and potentially malleable mechanism to help reduce alcohol consumption and related harms.
